# A role of CB1R in inducing θ-rhythm coordination between the gustatory and gastrointestinal insula

**DOI:** 10.1038/srep32529

**Published:** 2016-09-01

**Authors:** Youngnam Kang, Hajime Sato, Mitsuru Saito, Dong Xu Yin, Sook Kyung Park, Seog Bae Oh, Yong Chul Bae, Hiroki Toyoda

**Affiliations:** 1 Department of Neuroscience and Oral Physiology, Osaka University Graduate School of Dentistry, Suita, Osaka 565-0871, Japan; 2 Department of Oral Physiology, Graduate School of Medical and Dental Sciences, Kagoshima University, Kagoshima 890-8544, Japan; 3 Department of Oral Anatomy and Neurobiology, BK21, School of Dentistry, Kyungpook National University, Daegu 700-412, Republic of Korea; 4 Department of Neurobiology and Physiology, School of Dentistry, Seoul National University, Seoul 110-749, Republic of Korea

## Abstract

Anandamide (AEA) and *N*-oleoylethanolamine (OEA) are produced in the intestine and brain during fasting and satiety, respectively. Subsequently, AEA facilitates food intake via activation of cannabinoid type-1 receptors (CB1Rs) while OEA decreases food intake via activation of peroxisome proliferator-activated receptor-α (PPARα) and/or G-protein-coupled receptor 119 (GPR119). Neuronal activity in the gastrointestinal region of the autonomic insula (GI-Au-I) that rostrally adjoins the gustatory insula (Gu-I) increases during fasting, enhancing appetite while umami and sweet taste sensations in Gu-I enhances appetite in GI-Au-I, strongly suggesting the presence of a neural interaction between the Gu-I and GI-Au-I which changes depending on the concentrations of AEA and OEA. However, this possibility has never been investigated. In rat slice preparations, we demonstrate with voltage-sensitive dye imaging that activation of CB1Rs by AEA induces θ-rhythm oscillatory synchronization in the Gu-I which propagates into the GI-Au-I but stops at its caudal end, displaying an oscillatory coordination. The AEA-induced oscillation was abolished by a CB1R antagonist or OEA through activation of GPR119. Our results demonstrate that the neural coordination between the Gu-I and GI-Au-I is generated or suppressed by the opposing activities between CB1R and GPR119. This mechanism may be involved in the feeding behavior based on taste recognition.

The concentration of anandamide (AEA) and that of *N*-oleoylethanolamine (OEA) in the small intestine rises and falls, respectively, during fasting and vice versa upon refeeding^1^,^2^. AEA is concomitantly increased in the various brain regions^3^,^4^ during fasting, and subsequently facilitates food intake via activation of cannabinoid type 1 receptors (CB1Rs) while central administration of OEA decreases food intake via activation of peroxisome proliferator-activated receptor-α (PPARα) and/or G-protein-coupled receptor 119 (GPR119)^5^. In the rat insular cortex where CB1Rs are expressed^6^, the primary gustatory insula (Gu-I) in which chemo-sensory sensations from taste cells in the tongue are integrated as a taste recognition caudally adjoins the gastrointestinal autonomic insula (GI-Au-I) in which mechano-sensory and chemo-sensory sensations from the gastrointestinal tract^7^ are integrated as either hunger or satiety sensation. The neuronal activity in the GI-Au-I increases in the hunger state^8^, enhancing appetite while it decreases in response to feeding^9^. In contrast, neuronal activity induced in the Gu-I in response to tastant stimulation increases during fasting^10^ or decreases during satiety^11^. The appetite can be enhanced by the umami^12^ and sweet^13^ taste sensations, similar to the effect of appetizer^14^. On the contrary, the losses or decreases in taste sensation lead to a poor appetite^15^. Then, the enhancement of appetite would be brought about by the modulation of the neural activity in the GI-Au-I through the recognition of umami or sweet taste in the Gu-I, suggesting the presence of a neural interaction between the Gu-I and GI-Au-I that changes depending on the concentration of AEA or OEA. However, it has not been addressed whether AEA can cause a neural interaction between the Gu-I and GI-Au-I.

In the present study, we investigated whether and how AEA could induce neural coordination between the Gu-I and GI-Au-I in a slice preparation, using voltage-sensitive dye imaging. We found that an application of AEA induces θ-rhythm oscillatory excitation in the Gu-I which propagated into the GI-Au-I, displaying an oscillatory coordination between the Gu-I and GI-Au-I. The AEA-induced oscillation was abolished by a CB1R antagonist and OEA. A GPR119 agonist but not a PPARα agonist abolished the AEA-induced oscillation, and a GPR119 antagonist but not a PPARα antagonist blocked the inhibitory effects of OEA. The oscillatory coordination was found to be modulated by GABA_B_ receptor (GABA_B_R)-mediated feed-forward lateral inhibition. Taken together, the θ-rhythm neural coordination between the Gu-I and GI-Au-I modulated by the opposing activities between the CB1R and GPR119 may mediate the neural activities in the Gu-I and GI-Au-I during satiety or hunger.

## Results

### AEA induces oscillatory optical responses in the insular cortex

**Figure 1:**
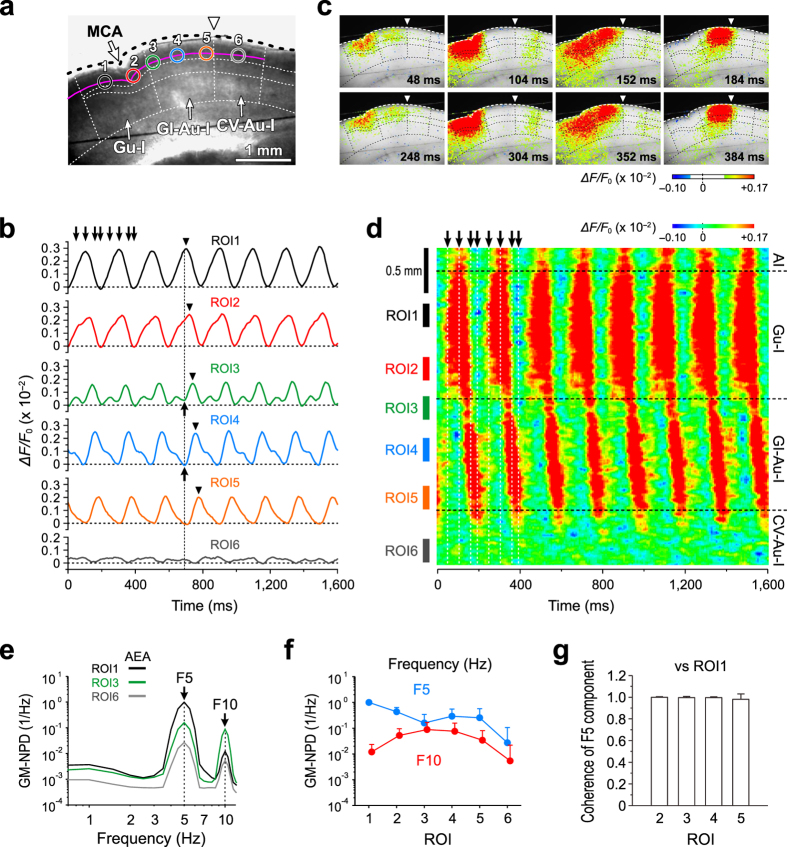
θ-rhythm oscillatory coordination between the Gu-I and GI-Au-I following AEA application. (**a**) A bright-field image of a slice of the insular cortex. The colored circles placed on a pink solid line drawn along L3 represent ROIs1-6. ⇓: middle cerebral artery (MCA). ∇: boundary between the GI-Au-I and CV-Au-I. (**b**) Sample traces of the temporal profiles at ROIs1–6 obtained following 3 μM AEA application. ↓: time points at which the respective pseudocolor images (**c**) were obtained. ↑: apparent inhibition. ▼: positive peaks. (**c**) Sample pseudocolor images of optical responses following AEA application. ∇: boundary between the GI-Au-I and CV-Au-I. (**d**) A line profile measured along L3 (a pink solid line in (**a)**) following AEA application. (**e**) GM-NPDs of the oscillatory responses at ROI1, ROI3 and ROI6 following AEA application (n = 16). (**f**) GM-NPDs at F5 and F10 of AEA-induced oscillation (n = 16). (**g**) The coherences of F5 component between the optical responses at ROI1 and ROIs2–5 (n = 16).

We first examined whether and how activation of the CB1R by AEA could induce the coordination of neural activity between the Gu-I and GI-Au-I in a slice preparation (Fig. 1a, see Methods). Optical signals are represented as pseudocolor images. In case of oscillatory optical responses, pseudocolor images were reconstructed by setting the zero baseline level to be the mean level of the bottom peaks of optical signals at respective pixels because there was no resting state in these oscillatory propagating optical responses (e.g. Fig. 1b). Following application of 3 μM AEA, excitatory optical responses shown as pseudocolor images emerged in the Gu-I and then propagated caudally into the GI-Au-I but stopped at its caudal end, which was repeated periodically at 5 Hz (Fig. 1c and Supplementary Movie 1).

To analyze the spatio-temporal profiles, we expediently placed six regions of interest (ROIs) along layer 2/3 (L2/3); ROIs1–2 in the Gu-I, ROI3 on the boundary between the Gu-I and GI-Au-I, ROIs4–5 in the GI-Au-I, and ROI6 in the cardiovascular region of autonomic insula (CV-Au-I) (Fig. 1a). The inter-center distance between respective ROIs was set to be larger than 400 μm, which is a diameter of a column-like cell assembly in the Gu-I found in our previous study^16^. The presence of such a functional column may be consistent with the gustotopy in the Gu-I^17^. The excitation initiated at ROIs1–2 propagated caudally to ROI5 (Fig. 1c) with a progressive shift of the peak of the optical responses (▼) as revealed in the respective temporal profiles (Fig. 1b). As the excitation propagated more caudally from ROI1 to ROI5, an apparent inhibition seemed to progress (Fig. 1b, ↑ and interrupted line) before the optical signals reached their respective peaks, likely attenuating the excitation and consequently causing delays in the respective peaks (Fig. 1b, ▼). To visualize the spatio-temporal profiles of the propagation of oscillatory optical responses along L3 from the Gu-I into the GI-Au-I, a line profile along L3 was constructed. The line profile clearly showed that the excitatory response emerged periodically at 5 Hz at ROIs1–2 (Gu-I), each of which propagated along L3 caudally into ROIs4–5 (GI-Au-I) through ROI3 with a progressive delay, and stopped around the caudal end of GI-Au-I but did not invade into the CV-Au-I including ROI6 (Fig. 1d).

Power spectral analysis of the temporal profiles of optical responses at ROI1, ROI3 and ROI6 representing respectively the Gu-I, GI-Au-I and CV-Au-I revealed that the oscillation was mainly composed of 5 Hz (F5) and 10 Hz (F10) frequency components (Fig. 1e). Consistent with the power spectral analysis, AEA induced sustained spike firings at θ-rhythm (ranged from 5 to 10 Hz, n = 6) in L3 pyramidal cells (PCs) in the Gu-I (Fig. S1). As shown in the spatial profile of optical responses represented by the geometric mean (GM) values of normalized power densities (NPDs) of F5 and F10 components at ROIs1–6 (Fig. 1f), ROI3 displayed the smallest GM value of F5 NPD (GM-F5-NPD) but the largest GM-F10-NPD among ROIs1–5. This is consistent with the temporal profile at ROI3 with the smallest amplitude and shortest duration (Fig. 1b). Although the temporal profiles at ROIs1–5 were very different and the progressive shifts of the peaks of the optical responses were observed from ROI1 to ROI5, the coherences of F5 component between the optical responses in ROI1 and other ROIs were almost 1 (Fig. 1g), revealing a very high correlation between those F5 components. These results suggest that the propagation of optical response from the Gu-I to the GI-Au-I was attenuated around its boundary, probably due to the presumed inhibition that would decrease the F5 power density (F5-PD) but increase the F10-PD at ROI3. A similar propagation of θ-oscillation with increasing phase shift in the hippocampus was reported based on the observation of local field potential recordings using multiple electrodes^18^.

We next examined whether and how GABA_A_ and/or GABA_B_ inhibitions are involved in the attenuation and the delay in the propagation of the AEA-induced oscillatory excitation. Of GABA_A_ and GABA_B_ inhibitions, GABA_B_ inhibition more markedly modulated the AEA-induced oscillatory optical response while GABA_A_ inhibition did not significantly modulate that, as described below.

### Involvement of GABA_B_R in the feed-forward inhibition

**Figure 2:**
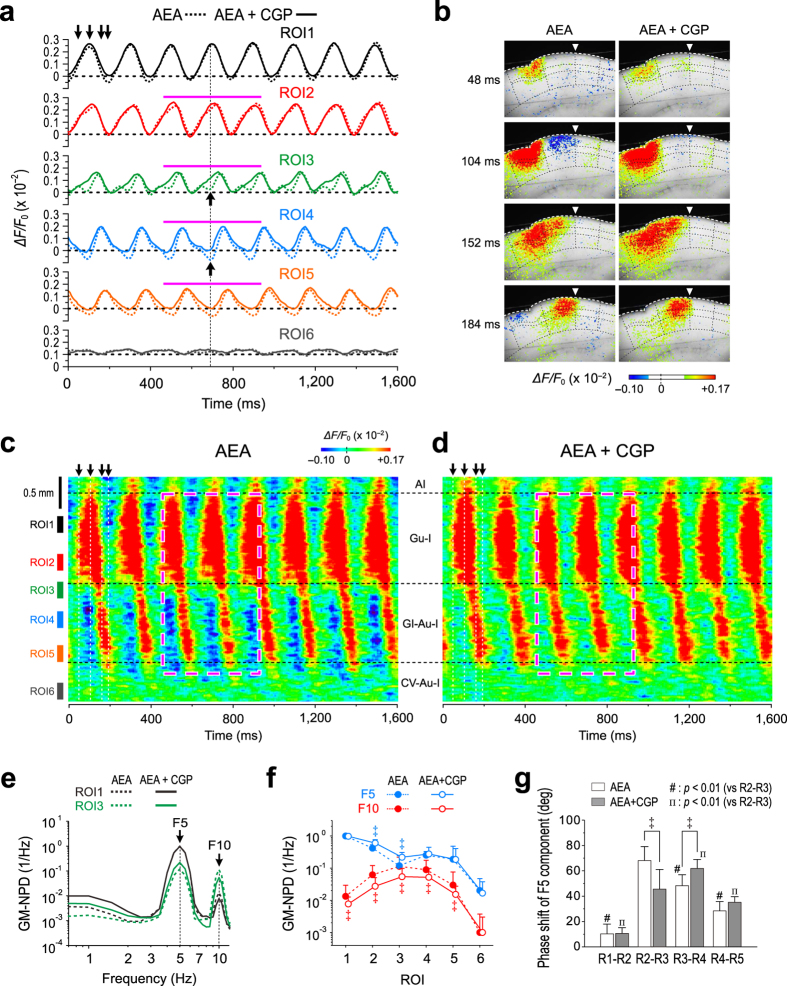
Resetting the baseline level of optical responses by referring to those obtained after CGP application. (**a**) Superimposed traces of the temporal profiles at ROIs1–6 obtained following application of 3 μM AEA alone (dotted lines) and following addition of 10 μM CGP55845 (CGP) (solid lines). (**b**) Reconstructed sample pseudocolor images of optical responses following application of AEA alone and those following addition of CGP. (**c**,**d**) A line profile measured along L3 following application of AEA alone (**c**) and that following addition of CGP (**d**). (**e**) GM-NPDs of the responses at ROI1 and ROI3 following application of AEA alone and those following addition of CGP (n = 5). (**f**) GM-NPDs at F5 and F10 of AEA-induced optical responses obtained before and after application of CGP (n = 5). ^‡^*p* < 0.01. (**g**) Phase shifts of F5 components between the two adjacent ROIs across ROIs1–5 obtained before and after application of CGP. ^‡^*p* < 0.01. ^‡(#)^*p* < 0.01 (vs R2-R3). ^‡(Π)^*p* < 0.01 (vs R2-R3).

CGP55845 (CGP)-sensitive inhibitory components may be contained in the negative or depressed components of oscillatory waves of optical responses, which is reflected in the valley or the attenuation during the rising phase of oscillation, respectively (Fig. 1b, ↑ and interrupted line). Therefore, based on the assumption that the top peak is not depressed by GABA_B_ action, the waves obtained before and after application of CGP were superimposed by aligning their top peak levels of the respective waves (e.g. at ROIs1–6) (Fig. 2a). Following addition of 10 μM CGP, the rising and falling phases of the respective waves of AEA-induced oscillatory responses at ROIs2–5 became much simpler, and the negative components of the optical responses at ROIs4–5 were considered to be largely abolished in view of the bottom peak level obtained after CGP application (Fig. 2a, ↑ and interrupted line). Thus, the bottom peak level of those obtained after CGP application was regarded as the zero baseline level for the control waves (e.g. at ROIs1–6) obtained before CGP application (Fig. 2a, see Methods) to reconstruct the pseudocolor images of the control optical response. This reconstruction disclosed the spatio-temporal profile of GABA_B_R-mediated feed-forward inhibition at the region over the ROIs3–5 in the control optical responses (Fig. 2b, left column and Supplementary Movie 2). However, despite the abolishment of GABA_B_R-mediated feed-forward inhibition by CGP, the oscillatory excitation did not propagate into the CV-Au-I including ROI6 (Fig. 2b, right column and Supplementary Movie 3).

The AEA-induced oscillatory excitation that emerged first in L2/3 and secondarily in L5 of the Gu-I propagated mostly into L2/3 but not prominently into L5 of the GI-Au-I (Fig. 1c). Poor excitation in L5 of the Gu-I and subsequent poor propagation into L5 of the GI-Au-I are revealed by the temporal and line profiles in L5 together with the spatial profile of GM-F5-NPD (Fig. S2). The presence of GABA_B_R-mediated feed-forward inhibition prior to the propagation into L2/3 of the GI-Au-I and its suppression by CGP were more clearly demonstrated in the L3 line profile images obtained before and after addition of CGP to the bath solution containing AEA (Fig. 2c,d). The CGP-sensitive inhibitory optical responses periodically emerged at ROIs3–5 but not at ROI6, prior to the propagation of the excitatory optical response into ROIs3–5 (GI-Au-I). These results clearly indicate that the excitation which emerged periodically at ROIs1–2 caused the GABA_B_R-mediated feed-forward lateral inhibition at ROIs3–5. Thus, it is likely that AEA induced the neural coordination between the Gu-I and GI-Au-I, which can be modulated by the feed-forward lateral inhibition mediated by GABA_B_R.

Although CGP did not significantly (^‡^*p* > 0.6) change GM-F5-NPDs at ROI1, ROI4 and ROI5, it significantly (^‡^*p* < 0.04) increased the GM-F5-NPDs at ROIs2‒3 (Fig. 2e,f). This result was consistent with the following analysis. Before application of CGP, the GM-F5-NPD at ROI3 was significantly (^‡^*p* < 0.02) smaller than those at ROIs1‒2 and ROIs4‒5. However, after application of CGP, the GM-F5-NPD at ROI3 was not significantly (^‡^*p* > 0.1) smaller than those at ROIs4‒5 whereas that remained significantly (^‡^*p* < 0.01) smaller than those at ROIs1‒2. In contrast, CGP significantly (^‡^*p* < 0.01) decreased the GM-F10-NPDs throughout ROIs1–5. Taken together, these observations suggest that GM-F10-NPDs reflect GABA_B_R-mediated inhibition more strongly compared to GM-F5-NPDs, as CGP made each wave broader to consequently decrease GM-F10-NPDs at ROIs1‒5 and subsequently increased GM-F5-NPDs only at ROIs2‒3. The reason why GM-F5-NPDs were increased only at ROIs2‒3 may be that the GABA_B_R-mediated inhibition occurred during the rising phase of the θ-rhythm oscillatory optical responses at ROIs2‒3 but not at other ROIs. These results suggest that GABA_B_R-mediated inhibition caused by the excitation in the Gu-I can differentially attenuate the propagation of the optical response beyond the boundary region (ROI3) into the GI-Au-I (ROIs4–5). These findings and the interpretation are consistent with those obtained by the analysis of the phase shift of F5 component along ROIs1–5. There was the largest and significant phase shift of F5 component between ROI2 and ROI3 (^‡^*p* < 0.01), compared to those between the two adjacent ROIs (Fig. 2g, open columns). CGP significantly (^‡^*p* < 0.01) decreased the phase shift of F5 components between ROI2 and ROI3, and consequently increased that between ROI3 and ROI4 significantly (^‡^*p* < 0.01) (Fig. 2g). Thus, the CGP-sensitive feed-forward inhibition causes a delay in the excitation propagation from ROI2 to ROI3 to synchronize the activities of PCs within the GI-Au-I. Taken together, these findings suggest that the neural coordination between the Gu-I and GI-Au-I can be modulated by GABA_B_R-mediated feed-forward lateral inhibition.

### GABA_A_ inhibition is not involved in the AEA-induced oscillation

**Figure 3:**
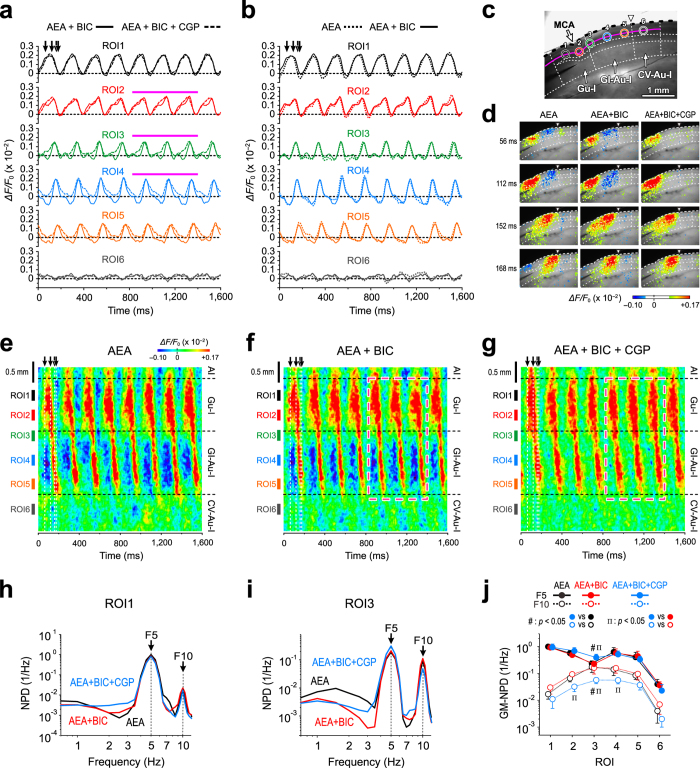
Involvement of GABA_B_R in the feed-forward inhibition of AEA-induced oscillatory optical responses. (**a**) Superimposed traces of the temporal profiles at ROIs1–6 obtained following co-application of 3 μM AEA and 10 μM BIC (solid lines) and after addition of 10 μM CGP (interrupted lines). ↓: time points at which the respective pseudocolor images (**d**) were obtained. (**b**) Superimposed traces of temporal profiles at ROIs1–6 obtained following application of AEA alone (dotted lines) and after addition of BIC (solid lines). (**c**) A bright-field image of the insular cortex in the horizontal slice. The colored circles represent ROIs1–6. ⇓: MCA. ∇: boundary between the GI-Au-I and CV-Au-I. (**d**) Reconstructed sample pseudocolor images of the optical responses following application of AEA alone (left column), those following co-application of AEA and BIC (middle column) and those following co-application of AEA, BIC and CGP (right column). (**e**–**g**) Line profiles measured along L3 following application of AEA alone (**e**), following co-application of AEA and BIC (**f**) and following co-application of AEA, BIC and CGP (**g**). (**h**,**i**) NPDs of the responses, shown in (**a**,**b**), at ROI1 (**h**) and ROI3 (**i**) following application of AEA alone, following co-application of AEA and BIC and following co-application of AEA, BIC and CGP. (**j**) GM-NPDs at F5 and F10 of the responses in ROIs1–6 following application of AEA alone (n = 4), following co-application of AEA and BIC (n = 4) and following co-application of AEA, BIC and CGP (n = 3). ^‡(#)^*p* < 0.05. ^‡(Π)^*p* < 0.05.

We previously demonstrated that 10 μM bicuculline markedly enhanced the optical responses evoked by stimulation of L4 in the Gu-I by blocking GABA_A_ receptors (GABA_A_Rs)^16^. Nevertheless, in contrast to the effect of CGP, the AEA-induced 5 Hz oscillatory optical responses were not affected by 10 μM bicuculline as revealed by the temporal and line profiles together with the power spectral analysis of optical responses (Fig. 3a,b,d–f,h–j). However, application of CGP in addition to bicuculline largely changed the optical responses, consistent with the results shown in Fig. 2. The pseudocolor images of optical responses were reconstructed by resetting the baseline level in the same way as was done in Fig. 2 (see Methods). Application of 10 μM CGP in addition to bicuculline largely changed the temporal profiles at ROIs3–5 (Fig. 3a) and removed the inhibition as demonstrated in a series of static pseudocolor images (Fig. 3d) and in the line profiles (Fig. 3f,g) although bicuculline did not affect the oscillatory optical responses (Fig. 3d–g). Subsequently, the GM-F5-NPD at ROI3 in the boundary between the Gu-I and GI-Au-I was significantly (^‡^*p* < 0.05, n = 3) increased following application of CGP in the presence of bicuculline, as represented by the change in the GM-F5-NPD at ROI3 from 0.23 × 1.2^±1^ to 0.39 × 1.1^±1^ (Fig. 3j). These findings indicate that the disinhibition of GABA_B_ action with CGP significantly affected the AEA-induced 5 Hz oscillatory optical response.

### AEA induces oscillatory synchronization in the Gu-I by activation of CB1R and its inhibition by OEA

**Figure 4:**
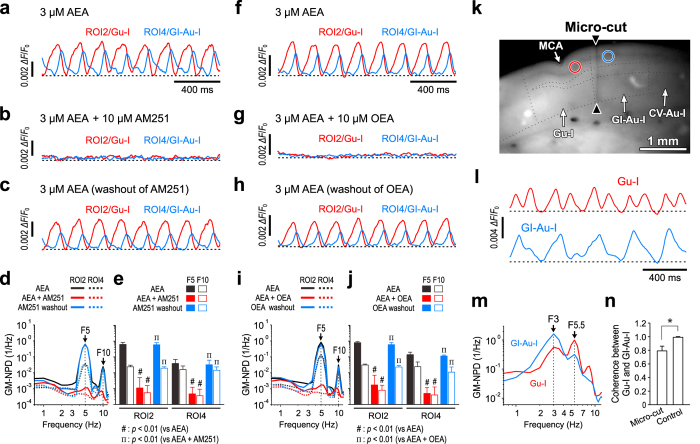
Effects of AM251/OEA on AEA-induced oscillatory coordination and its disruption by micro-cut. (**a–c**, **f–h**) Superimposed traces of the temporal profiles of AEA-induced responses at ROI2 and ROI4 obtained before and after application of 10 μM AM251 (**a**,**b**, respectively) and 10 μM OEA (**f**,**g**, respectively), and those obtained 30 min after washout of AM251 (**c**) and OEA (**h**). (**d**,**i**) GM-NPDs of the AEA-induced responses at ROI2 and ROI4 obtained before and after application of AM251 (**d**) and OEA (**i**), and those obtained after washout of AM251 (**d**) and OEA (**i**). (**e**) GM-F5-NPDs and GM-F10-NPDs : AEA/ROI2, 0.6232 × 1.4^±1^ and 0.0240 × 1.3^±1^; AEA + AM251/ROI2, 0.0011 × 4.5^±1^ and 0.0006 × 1.9^±1^; AEA(AM251 washout)/ROI2, 0.5954 × 1.3^±1^ and 0.0197 × 1.2^±1^; AEA/ROI4, 0.0389 × 1.8^±1^ and 0.0161 × 1.7^±1^; AEA + AM251/ROI4, 0.0005 × 2.6^±1^ and 0.0004 × 2.5^±1^; AEA(AM251 washout)/ROI4, 0.0322 × 1.9^±1^ and 0.0145 × 1.6^±1^ (n = 5). ^†(#)^*p* < 0.01 (vs AEA). ^†(Π)^*p* < 0.01 (vs AEA + AM251). (**j**) GM-F5-NPDs and GM-F10-NPDs: AEA/ROI2, 0.8087 × 1.1^±1^ and 0.0310 × 1.2^±1^; AEA + OEA/ROI2, 0.0017 × 4.2^±1^ and 0.0008 × 2.0^±1^; AEA(OEA washout)/ROI2, 0.5959 × 1.4^±1^ and 0.0234 × 1.2^±1^; AEA/ROI4, 0.1402 × 1.4^±1^ and 0.0243 × 2.0^±1^; AEA + OEA/ROI4, 0.0006 × 2.3^±1^ and 0.0004 × 3.1^±1^; AEA(OEA washout)/ROI4, 0.1147 × 1.2^±1^ and 0.0110 × 2.1^±1^ (n = 5). ^†(#)^*p* < 0.01 (vs AEA). ^†(Π)^*p* < 0.01 (vs AEA + OEA). (**k**) A resting-light-intensity image of the insular cortex in a micro-cut slice. The colored circles represent ROIs in the Gu-I (red) and the GI-Au-I (blue). ⇓: MCA. Black facing arrowheads indicate micro-cut throughout all layers at the rostral end of the GI-Au-I. (**l**) Sample traces of the temporal profiles of AEA-induced responses at the Gu-I and GI-Au-I in a micro-cut slice. (**m**) GM-NPDs of the responses at the Gu-I and the GI-Au-I in micro-cut slices (n = 5). (**n**) The mean value of the coherence of F5.5 component between the oscillations in the Gu-I and GI-Au-I in the micro-cut slice (0.792 ± 0.075, n = 5) and that of the coherence of F5 component in the intact slice (0.990 ± 0.008, n = 17). *p < 0.01.

We next examined the effects of AM251 as an antagonist of the CB1R and those of OEA as an agonist of either the GPR119 or PPARα on the AEA-induced oscillation in the Gu-I and the subsequent neural coordination between the Gu-I and GI-Au-I. AEA-induced oscillatory synchronization in the Gu-I and their subsequent propagation into the GI-Au-I (Fig. 4a,f) were almost completely abolished by addition of 10 μM AM251 (Fig. 4b) or 10 μM OEA (Fig. 4g). Following washout of AM251 or OEA for > 20 min, the oscillatory coordination recovered considerably (Fig. 4c,h). The power spectral analysis of optical responses at ROI2 and ROI4 revealed that the GM-F5-NPDs and GM-F10-NPDs obtained following application of AEA were significantly (^†^*p* < 0.01, ^†^*p* < 0.01, respectively) decreased by addition of AM251 (Fig. 4d,e, n = 5) and OEA (Fig. 4i,j, n = 5), and thereafter significantly (^†^*p* < 0.01, ^†^*p* < 0.01, respectively) increased following washout of AM251 (Fig. 4d,e, n = 5) or OEA (Fig. 4i,j, n = 5). These results suggest that the oscillation in the Gu-I was induced through activation of the CB1R while it was abolished through activation of either the GPR119 or PPARα. Subsequently, the AEA-induced oscillatory coordination between the Gu-I and GI-Au-I was abolished by AM251 or OEA as reflected in significant decreases in the coherence of F5 component between ROI2 and ROI4 from 0.989 ± 0.007 to 0.456 ± 0.427 following application of AM251 (**p < 0.05; n = 5) and from 0.999 ± 0.001 to 0.549 ± 0.100 following application of OEA (**p < 0.01; n = 5).

We next investigated whether there is any positive feedback from the GI-Au-I to the Gu-I by using micro-cut slices and caudally tilted-up ones (see Methods). When the connection between the Gu-I and GI-Au-I was disrupted by making a cut throughout the layers 1‒6 at the caudal end of the Gu-I or at the rostral end of the GI-Au-I (Fig. 4k), AEA induced a less regular 5‒6 Hz oscillation in the Gu-I and a less regular 3 Hz oscillation in the GI-Au-I in contrast to the intact slices (Fig. 4l). This result indicates that each 5‒6 Hz wave was generated in the Gu-I but did not propagate into the GI-Au-I. Furthermore, the finding that NPD at F5.5 was significantly (*p < 0.02) larger in the Gu-I than in the GI-Au-I whereas NPD at F3 was significantly (*p < 0.01) larger in the GI-Au-I than in the Gu-I (Fig. 4m, n = 5) clearly reveals that there is no apparent propagation of oscillatory waves from the Gu-I to the GI-Au-I. In consistent with this finding, the coherence of F5.5 component between the oscillations in the Gu-I and GI-Au-I in the micro-cut slices (n = 5) was significantly (*p < 0.01) smaller than that of F5 component in intact slices (n = 17) (Fig. 4n). These results clearly indicate that F5 component of AEA-induced oscillation was generated in the Gu-I and the subsequent propagation of F5 component from the Gu-I to the GI-Au-I was largely abolished by the micro-cut throughout the layers 1–6. We also found the absence of the positive feedback from the GI-Au-I to the Gu-I by using caudally tilted-up slices (Fig. S3a–d). This was consistent with the observation that neither oscillation nor propagation was induced by AEA in the slices cut at 700‒1050 μm more dorsally from the RF in which neither the Gu-I nor the GI-Au-I was included (Fig. S3e,f). Taken together, it is strongly suggested that the AEA induced the oscillation in the Gu-I, which subsequently propagated into the GI-Au-I without receiving positive feedback from there.

### Not PPARα but GPR119 is involved in the inhibitory effects of OEA on the AEA-induced oscillation

**Figure 5:**
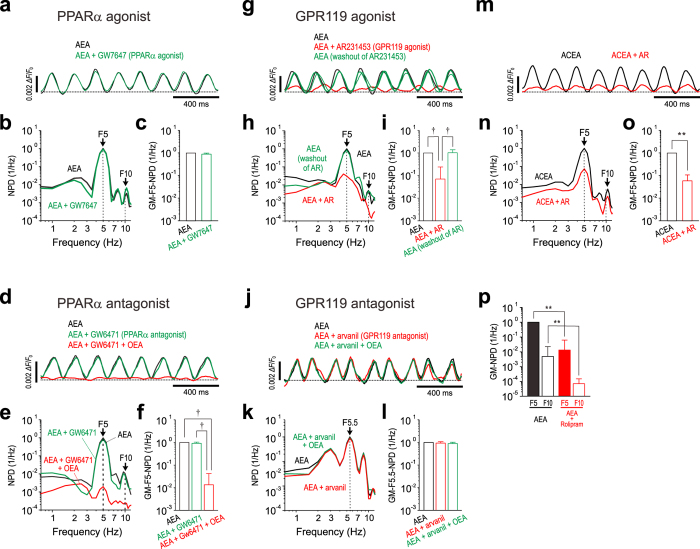
Involvement of GPR119 but not PPARα in the inhibitory effects of OEA on the AEA-induced oscillation. (**a**,**b**) Sample traces of the temporal profiles of the AEA-induced responses in Gu-I (**a**) and their NPDs (**b**) obtained before and 30 min after application of 1 μM GW7647. The power spectral density obtained after application of GW7647 was normalized to the F5-PD of the control. (**c**) GM-F5-NPDs (n = 5): AEA, 1; AEA + GW7647, 0.886 × 1.1^±1^. (**d,e**) Sample traces of the temporal profiles of the AEA-induced responses in Gu-I (**d**) and their NPDs (**e**) obtained before, 30 min after application of 10 μM GW6471 and further 30 min after co-application of GW6471 and 10 μM OEA. (**f**) GM-F5-NPDs (n = 5): AEA, 1; AEA + GW6471, 0.897 × 1.2^±1^; AEA + GW6471 + OEA, 0.009 × 2.5^±1^. ^†^*p* < 0.01. (**g**,**h**) Sample traces of the temporal profiles of the AEA-induced responses in Gu-I (**g**) and their NPDs (**h**) obtained before, after application of 1 μM AR231453 (AR) and further after washout of AR. (**i**) GM-F5-NPDs (n = 5): AEA, 1; AEA + AR, 0.071 × 3.4^±1^; AEA (after washout of AR), 1.029 × 1.4^±1^. ^†^*p* < 0.01. (**j**,**k**) Sample traces of the temporal profiles of the AEA-induced responses in Gu-I of the micro-cut slice (**j**) and their NPDs (**k**) obtained before, 20 min after application of 50 μM arvanil and further 20 min after co-application of arvanil and 10 μM OEA. (**l**) GM-NPDs at F5.5 (n = 5): AEA, 1; AEA + arvanil, 0.930 × 1.2^±1^; AEA + arvanil + OEA, 0.889 × 1.1^±1^. (**m**,**n**) Sample traces of the temporal profiles of the 1 μM ACEA-induced response in Gu-I (**m**) and their NPDs (**n**) obtained before and after addition of 1 μM AR. (**o**) GM-F5-NPD (n = 4): ACEA, 1; ACEA + AR, 0.059 × 1.8^±1^. **p < 0.01. (**p**) Significant decreases in GM-NPDs at F5 and F10 in Gu-I following addition of 10 μM rolipram to the bath solution containing 3 μM AEA (n = 7). **p < 0.01.

We next examined a possible involvement of PPARα in the abolishment of the AEA-induced oscillation by OEA using an agonist of PPARα, GW7647 (EC_50_ = 6 nM)^19^ and an antagonist of PPARα, GW6471 (IC_50_ = 0.24 μM)^20^. The AEA-induced oscillation in the Gu-I was not affected by 10 μM GW7647 (Fig. 5a,b), as also revealed by no significant decrease in the GM-F5-NPD (^**^p > 0.3) (Fig. 5c, n = 5). Furthermore, 1 μM GW6471 did not significantly affect the AEA-induced oscillation in the Gu-I (Fig. 5d,e) as also revealed by no significant difference in the GM-F5-NPD (^†^*p* > 0.7) (Fig. 5f, n = 5). However, the subsequent application of 10 μM OEA in the presence of GW6471 markedly suppressed the AEA-induced oscillation (Fig. 5d,e) as also revealed by the significant decrease in the GM-F5-NPD (AEA vs AEA + GW6471 + OEA, ^†^*p* < 0.01; AEA + GW6471 vs AEA + GW6471 + OEA, ^†^*p* < 0.01) (Fig. 5f, n = 5). These results clearly indicate that PPARα is not involved in the OEA-mediated inhibition of the AEA-induced oscillation in the Gu-I.

Because the effects of OEA are mediated by the activity of either PPARα or GPR119^21^,^22^, it was investigated whether GPR119 mediates the inhibitory effects of OEA on the AEA-induced oscillation. Accordingly, we examined effects of an agonist and an antagonist of GPR119, AR231453 (EC_50_ = 4.7 nM)^23^ and arvanil^24^, respectively, on the AEA-induced oscillation. The AEA-induced oscillation in the Gu-I was markedly attenuated by applying 1 μM AR231453 in the presence of 3 μM AEA (Fig. 5g,h) as also revealed by the significant decrease in the GM-F5-NPD (AEA vs AEA + AR; ^†^*p* < 0.01, n = 5) (Fig. 5i). After washout of AR231453 for > 20 min, the oscillation was restored almost completely (Fig. 5g,h) as also revealed by the GM-F5-NPD (AEA vs AEA after washout of AR; ^†^*p* > 0.9, AEA + AR vs AEA after washout of AR; ^†^*p* < 0.01, n = 5) (Fig. 5i). The AEA-induced oscillation in the Gu-I was not significantly changed by 50 μM arvanil (Fig. 5j,k) as also revealed by the GM-NPD at F5.5 (^†^*p* > 0.28) (Fig. 5l, n = 5). The subsequent application of OEA in the presence of arvanil had almost no effect on the AEA-induced oscillation (Fig. 5j,k) as also revealed by the GM-NPD at F5.5 (AEA + arvanil vs AEA + arvanil + OEA; ^†^*p* > 0.49, AEA vs AEA + arvanil + OEA; ^†^*p* > 0.10, n = 5) (Fig. 5l). These results would indicate that GPR119 but not PPARα is involved in the inhibitory effects of OEA on the AEA-induced oscillation in the Gu-I.

Finally, we confirmed that a more potent synthesized agonist of CB1R, ACEA (*K*_i_ = 1.4 nM)^25^ compared to AEA (*K*_i_ = 69 nM)^26^ induced the oscillation that can be suppressed by activation of GPR119 with AR231453. The oscillation induced by ACEA was suppressed by 1 μM AR231453 (Fig. 5m,n) as also revealed by the significant decrease in the GM-F5-NPD in the Gu-I (**p < 0.01) (Fig. 5o, n = 4). Taken together, these results strongly suggest that not PPARα but GPR119 is involved in the inhibitory effects of OEA on the oscillation induced in the Gu-I by activation of CB1R.

The production of cAMP is decreased by AEA through activation of the CB1R^27^ whereas it can be increased through the activation of GPR119 by OEA or its agonists in mammalian cells^21^,^28^ but not through activation of PPARα. Therefore, we next examined whether the downregulation of cAMP is involved in the AEA-induced oscillation. Rolipram (10 μM) that upregulates cAMP by suppressing cAMP hydrolysis^29^ abolished the AEA-induced oscillation as represented by the significant (**p < 0.01) decreases in the GM-F5-NPDs and GM-F10-NPDs in the Gu-I (Fig. 5p, n = 7). Taken together, it is strongly suggested that the θ-rhythm oscillation in the Gu-I is modulated by the opposing activities between CB1R and GPR119 through the downregulation and upregulation of cAMP, respectively.

## Discussion

We concluded that the oscillatory excitation was generated in the Gu-I by activation of CB1Rs, and subsequently propagated into the GI-Au-I based on the following four observations. Firstly, the GM-F5-NPD was significantly (**p < 0.04 and ^†^*p* < 0.04) larger at ROI2 (0.435 × 1.5^±1^) than at ROI4 (0.280 × 2.0^±1^) (Fig. 1f, n = 16). Secondly, the AEA-induced oscillation propagated invariably from the Gu-I into the GI-Au-I with a time delay (Figs 1d, 2c,d and 3e–g) while any oscillatory propagation from GI-Au-I to Gu-I was never observed (Figs 1, 2 and 3). Thirdly, when the connection between the Gu-I and GI-Au-I was disrupted by making a cut in slices (Fig. 4k), AEA induced different oscillations between the Gu-I and GI-Au-I (Fig. 4l). F5.5 component was dominant in the Gu-I but not in the GI-Au-I (Fig. 4m), suggesting that F5–6 component of AEA-induced oscillation was generated in the Gu-I and its subsequent propagation into the GI-Au-I was abolished by the cut in slices (see below). Fourthly, F5 oscillation was invariably induced in the Gu-I regardless of whether the GI-Au-I was included or not in the slices examined (Fig. S3a–d) while neither oscillation nor propagation was observed when examined in the dorsal slices in which the Gu-I was not included (Fig. S3e,f). Because the oscillation was thus generated in the Gu-I and propagated into the GI-Au-I but stopped at its caudal end without any further propagation into the CV-Au-I, there may be a neural coordination between the Gu-I and GI-Au-I.

Based on the following three reasons, it was possible to draw a conclusion that the micro-cut in slices abolished the propagation of F5–6 component of AEA-induced oscillation from the Gu-I to the GI-Au-I. Firstly, if the generation of F5–6 component in the Gu-I and F3 component in the GI-Au-I were caused by a propagation of oscillation from the Gu-I to the GI-Au-I despite the micro-cut, both the F5–6 and F3 components should have been larger in the Gu-I than in the GI-Au-I as was the case in the intact slice. Secondly, given the propagation of F5–6 component from the Gu-I to the GI-Au-I, F3 component would disappear in the GI-Au-I due to the propagation of higher firing activities that generate F5–6 component from the Gu-I into the GI-Au-I, as would be the case in the intact slices. The presence of F3 component in the GI-Au-I is incompatible with the propagation of F5–6 component from the Gu-I into the GI-Au-I unless the connection between the Gu-I and GI-Au-I is disrupted. Thirdly, the coherence of F5–6 component between the Gu-I and GI-Au-I was much smaller in the micro-cut slices than in the intact slices. Taken together, these observations and notions indicate that the propagation of F5–6 component of AEA-induced oscillation from the Gu-I to the GI-Au-I was abolished by the micro-cut in slices. Thus, the experiment in the micro-cut slice (Fig. 4k–n) revealed that the oscillatory coordination between the Gu-I and GI-Au-I is likely to be mediated by the cortico***-***cortical connections through axon collaterals of L3 PCs in the Gu-I and GI-Au-I, and that the network structure of the Gu-I may be different from that of the GI-Au-I because AEA induced different oscillations once the synaptic connections between the two areas were severed by the micro-cut.

The CB1R is coupled to G_i/o_ to downregulate cAMP production, and is also coupled positively to K^+^ and negatively to Ca^2+^ channels^27^. In the rat neocortex, the CB1R is predominantly localized to the axon terminals of cholecystokinin/calbindin-positive GABAergic interneurons^30^, in which CB1R activation leads to decreases in GABA release through the inhibition of Ca^2+^c hannel^31^. Excitatory synaptic transmission in L2/3 PCs in the neocortex was similarly inhibited through activation of the CB1R^32^,^33^. However, it has also been reported that cannabinoids increased glutamatergic transmission by activation of the CB1R in cortical neurons^34^, in which CB1R activation may have downregulated cAMP production rather than inhibiting presynaptic Ca^2+^ channels, because it is known that the upregulation of cAMP production decreases glutamate release in L5/6 PCs in the prefrontal cortex^35^. Conversely, it has been reported that downregulation of cAMP production by 5-HT_1A_ or adenosine A_1_ receptors resulted in the inhibition of GABA release^36^,^37^.

The present study demonstrated that application of AEA caused the oscillatory excitation in the Gu-I (Figs 1, 2, 3, 4 and 5) while AM251 abolished this oscillation (Fig. 4a–e), indicating that the AEA-induced oscillatory excitation in the Gu-I is mediated by the activation of CB1R. The abolishment of AEA-induced oscillation by rolipram that suppresses cAMP hydrolysis (Fig. 5p) was consistent with the generation of oscillation through the downregulation of cAMP production by activation of CB1R. Furthermore, OEA suppressed the AEA-induced oscillation definitely not through the activation of PPARα but through the activation of GPR119. This is because OEA suppressed the AEA-induced oscillation even in the presence of GW6471 (PPARα antagonist) (Fig. 5d–f) while neither GW7647 (PPARα agonist) nor GW6471 affected the AEA-induced oscillation (Fig. 5a–f). Furthermore, AR231453 (GPR119 agonist) abolished the AEA-induced oscillation (Fig. 5g–i), and arvanil (GPR119 antagonist) blocked the inhibitory effects of OEA (Fig. 5j–l). These observations also support the involvement of cAMP signal transduction in the AEA-induced oscillation because the cAMP level is increased by activation of GPR119^21^,^28^, consistent with the effects of rolipram as a phosphodiesterase type 4 inhibitor^29^.

When OEA was administered intraperitoneally^22^ or injected into the lateral hypothalamus^5^, OEA exerted the inhibitory effects on food intake by increasing the fatty acid metabolism and transport in liver and heart^38^ through activation of PPARα^22^. Thus, PPARα-mediated signal transduction caused by OEA appears to have no relevance to cortical information processing, and is distinct from cAMP signal transduction that is critically involved in the AEA-induced oscillation. The precise role of GPR119 in inhibiting the oscillation induced by activation of CB1R may be studied by using knockdown or knockout of GPR119 as a future study.

We found that in the Gu-I, the CB1R and GPR119 were expressed in the axon terminals of both non-GABAergic and GABAergic neurons (Fig. S4). Although both receptors were also expressed in dendritic spines (Fig. S4), their functional roles remain unknown. These results suggest that AEA-induced oscillatory synchronous excitation at 5 Hz in PCs in the Gu-I may have been caused partly by the facilitation of glutamatergic inputs onto PCs via activation of the CB1R coupled to G_i/o_ in the pre-terminals of neighboring PCs. The synchronous excitation may also have been partly caused by the disinhibition of PCs via activation of the CB1R in the pre-terminals of some GABAergic interneurons, given that the CB1R is coupled to G_i/o_ in these pre-terminals.

In the neocortex, parvalbumin-positive GABAergic interneurons that send long horizontal axons to form basket-like axon terminals on PCs^39^ lacked CB1R immunoreactivity at their axon terminals^30^. When excitation was caused in the Gu-I by AEA, parvalbumin-positive fast-spiking neurons in the Gu-I may have exerted GABA_B_R-mediated lateral inhibition (Figs 2 and 3). The lack of GABA_A_R involvement in the lateral inhibition (Fig. 3) was in agreement with a report showing that GABA_A_Rs at synapses made by fast-spiking neurons are blocked by endogenous and exogenous AEA^40^. Thus, the AEA-induced excitation in the Gu-I caused the GABA_B_R-mediated lateral inhibition, which attenuated the propagation of the optical response from the Gu-I to the GI-Au-I, thereby modulating the neural coordination between the Gu-I and GI-Au-I.

Until now, there are numerous studies investigating feed-forward inhibition. However, no study has ever demonstrated the spatio-temporal profiles of the feed-forward inhibition during on-going propagation of excitation or synchronization. The feed-forward inhibition has been simply considered to inhibit the on-going excitation of a neuronal population to limit the extent of excitation spread as lateral inhibition or to increase the precision of spike timing by narrowing the depolarization window as revealed by a curtailment of EPSP/EPSC by the subsequent IPSP/IPSC^41^. Thus, the relationship between the spatial and temporal profiles of feed-forward inhibition remained unclear. To our best knowledge, we have for the first time succeeded to visualize the feed-forward inhibition that is induced in the GI-Au-I by the θ-rhythm oscillatory excitation in the Gu-I prior to the propagation of respective excitation waves from the Gu-I to the GI-Au-I (Figs 2b–d and 3d–g). The subtraction between the temporal profiles at respective ROIs obtained before and after application of CGP (Fig. S5b,d, blue and red traces, respectively) revealed the GABA_B_R-mediated inhibitory components at respective ROIs, which were more prominent at ROIs3‒5 than at ROIs1‒2 (Fig. S5b,d, pink traces). The time-to-peak of the GABA_B_R-mediated inhibitory components was earliest at ROI2/3 but there were no marked delays in the time-to-peaks of the GABA_B_R-mediated inhibitory components between ROI2 and ROI4/5 (Fig. S5b,d, pink arrowheads) whereas the time-to-peak of the excitatory component delayed more largely as the excitation propagated caudally (Fig. S5b,d, red arrowheads), suggesting that the GABA_B_R-mediated inhibitory component was largely induced by feed-forward mechanism. Thus, the feed-forward inhibition precedes the excitation to curtail its rising phase of the optical responses in ROIs3‒5, consequently minimizing the delay in the propagation of excitatory optical responses from ROI3 to ROI5. Contrary to the previous reports on the feed-forward inhibition^41^, such a feed-forward inhibition neither limits the extent of excitation spread in L3 nor follows the excitation. These observations would provide a new concept of feed-forward lateral inhibition that is distinct from the concept of feed-forward inhibition reported in previous studies^41^. However, L3 PCs in the Gu-I may receive both local GABAergic projection in the Gu-I and long range GABAergic projection from GI-Au-I. Such a local inhibition within the Gu-I would not cause phase shift or propagation delay but rather contribute to synchronize the activity of PCs in the Gu-I while a possible long range GABAergic projection from the GI-Au-I to the Gu-I causing “feedback” inhibition in the Gu-I would modulate the oscillation. Indeed, CGP slightly decreased the bottom peak of the oscillation in the rostral Gu-I (ROI1; Fig. 2a–d) which increases as the excitation in the GI-Au-I progresses. Such a local or “feedback” inhibition was not so prominent as the feed-forward inhibition, as far as CGP-sensitivity is concerned (Figs 2 and 3). Thus, the feed-forward and feedback inhibitions were separately seen with different spatio-temporal profiles, and were not incompatible with each other.

It is possible that F10 components simply reflect a harmonic of the 5 Hz oscillations. However, this is unlikely for the following reasons. First, the results from the whole-cell current-clamp recordings demonstrated that AEA induced spike firings at approximately 10 Hz as well as 5 Hz in the L3 PCs of the Gu-I (Fig. S1), suggesting that F5 and F10 components of oscillatory optical responses are mediated by spike firings at 5 and 10 Hz induced by AEA in L3 PCs, respectively. Furthermore, as clearly seen in the temporal profiles of the optical responses especially at ROI3, the waveforms of the AEA-induced oscillations were slender due to the GABA_B_R-mediated inhibition, i.e., fast rising and fast falling (Fig. 1b, ROI3), whereas those became broader after CGP application (Fig. 2a, ROI3). Thus, the reduction of F10 components by CGP can be explained by the inhibitory effect of CGP on F10 components but not by reducing the non-sinusoidality of 5 Hz oscillations. Taken together, it is likely that the power at 10 Hz frequency is distinct from that reflecting a harmonic of the 5 Hz oscillation.

We have recently demonstrated that transient receptor potential vanilloid type 1 (TRPV1) activation in the Gu-I induces θ-band (4–8 Hz) synchronization between the Gu-I and Au-I^42^. This network coordination induced by TRPV1 activation could be responsible for autonomic responses to tasting and ingesting spicy foods. Activation of TRPV1 induced the oscillatory optical responses across the Gu-I and the whole area of the Au-I^42^, while the AEA-induced oscillations in the Gu-I propagated into GI-Au-I but not into the CV-Au-I (Figs 1, 2 and 3) although AEA is also known to be an agonist to TRPV1 receptors^43^. Therefore, the present study suggests that activation of CB1Rs by AEA may facilitate the food intake without causing cardiovascular and/or respiratory responses. It has recently been reported that tastant information is encoded as the phase and amplitude of 4–5 Hz frequency band of local field potentials in the Gu-I^44^ while the GI-Au-I is known to be involved in visceral autonomic controls depending on emotional states^45^. We therefore propose that the θ-rhythm oscillatory neural coordination between the Gu-I and GI-Au-I is a novel brain mechanism for inducing the appetite by hedonic taste sensation which is caused as a consequence of the neural integration between taste and appetite recognition in the Gu-I and the GI-Au-I, respectively.

## Methods

All experimental protocols were approved by the animal ethics committees of the Osaka University Graduate School of Dentistry and the Kyungpook National University for the care and use of laboratory animals, and all experiments were performed in accordance with the relevant guidelines.

### Slice preparation

**Figure 6:**
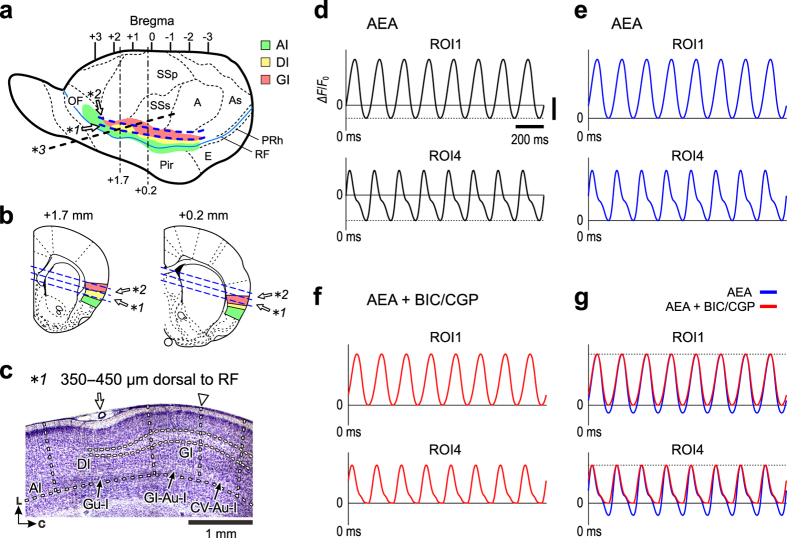
Cytoarchitecture of the insular cortex. (**a**) A lateral view of rat whole brain, in which cytoarchitectural differences of the insular cortex are colored differentially. Agranular insula (AI), green; dysgranular insula (DI), yellow; granular insula (GI), pink. Blue interrupted lines indicate the locations of the two serial tilted-horizontal slices, the bottom surface of the first slice (*1) was 350 μm dorsal to the rhinal fissure (RF) and that of the second slice (*2) was 700 μm dorsal to the RF. Black interrupted line indicates the location and orientation of the caudally tilted-up slice (*3). The blue continuous curve; RF. A, auditory cortex; As, association cortex; E, entorhinal cortex; OF, orbitofrontal cortex; Pir, piriform cortex; PRh, perirhinal cortex; SSp, primary somatosensory cortex; SSs, secondary somatosensory cortex. (**b**) Coronal sections obtained at +1.7 and +0.2 mm from bregma, in which the bottom and top surfaces of the two horizontal slices are shown with blue interrupted lines at respective bregma levels. (**c**) A Nissl-stained histological section (100 μm thickness) of the tilted horizontal slice at 350‒450 μm dorsal to the RF. Open arrow; middle cerebral artery (MCA). Open arrowhead; border between the GI-Au-I and CV-Au-I. (**d**) BV_Ana sets the signal intensities at all pixel subsets (3 × 3) equally to be zero at the time when the optical recording was started (the zero time point). Y-axis title and scale bars for y-axis (arbitrary units) and x-axis (200 ms) shown in (**d**) applies to all panels in (**e**‒**g**). (**e**) The mean values of the bottom peak levels of oscillatory optical signals at the respective pixel subsets were regarded as the zero baseline level of optical signals at respective pixels. (**f**) The bottom peak level of the oscillatory optical responses obtained after application of bicuculline/CGP was regarded as the real baseline level. (**g**) After defining the real baseline level (**f**), the waves obtained before and after application of bicuculline/CGP were superimposed by aligning the top peaks of waves (e.g. at ROI1 and ROI4) to determine the zero baseline level of the waves obtained before bicuculline/CGP application.

Wistar and Sprague-Dawley (SD) rats of both sexes at postnatal days 14–22 and 14–28, respectively, were used. Two serial slice sections, namely tilted-horizontal slices, were cut by referring to the middle cerebral artery (MCA) at 350 μm thick in parallel to the RF in a plane tilted up in the medial direction by 15° against the horizontal plane (Fig. 6a,b, *1 and *2). The cortical surfaces (layer 1) of the bottom sides of the first and second slices were 350 and 700 μm dorsal to the RF, respectively. To examine the cytoarchitecture of the insular cortex, seven serial sections of 100 μm thickness were cut at the levels between 350‒1050 μm dorsal to RF for Nissl staining. The first slice included the agranular insula (AI), the dysgranular insula (DI) and the granular insula (GI) as shown in the Nissl stained section cut at 350‒450 μm dorsal to the RF (Fig. 6c)^42^. The boundary between the DI and GI is located at the point where the thickness of L4 starts to become thinner, as the insular cortex shifts rostrally from the rostral end of the GI to the rostral end of the DI, and was found to be located between 500 and 750 μm caudal to the MCA (bregma + 1.0 and + 0.75 mm) (Fig. 6c)^42^. DI represents the gustatory area (Gu-I), and GI represents the autonomic area (Au-I)^7^. The boundary between the gastrointestinal region of the Au-I (GI-Au-I) and the cardiovascular region of the Au-I (CV-Au-I) is located between 1250 and 1500 μm caudal to the MCA (bregma + 0.25 and 0 mm) (Fig. 6c), and the caudal end of the Au-I is located at bregma −0.5 mm (≈2.0 mm caudal to the MCA)^7^. In the second slice, the DI was not included while the granular sensory cortex (GS; SSp, primary somatosensory cortex and SSs, secondary somatosensory cortex) and GI (Au-I) were included as shown in the Nissl stained section cut at 850‒950 μm dorsal to the RF (Fig. S3e). To examine whether there is any feed-forward or feedback interactions between neurons in the Gu-I and GI-Au-I, we used a micro-cut slice in which the connection between the Gu-I and GI-Au-I was disrupted by making a cut throughout the layers 1‒6 at the caudal end of the Gu-I or at the rostral end of the GI-Au-I (Fig. 4k). The micro-cut slice was also used in the experiment to examine whether a GPR119 antagonist can block the inhibitory effect of OEA on AEA-induced oscillations (Fig. 5j–l). As a further negative control experiment for the network coordination between the Gu-I and GI-Au-I, the slices were cut tangentially from the crossing point of the MCA and the RF to the caudo-dorsal area of the insular and somatosensory cortices, so that the dysgranular Gu-I was localized in the rostral one-third of such slices while the caudal two-third was mostly occupied by the somatosensory areas with a small area of GI-Au-I in its rostralmost part (Fig. 6a, black interrupted line, *3 and Fig. S3a). Such slices were termed as caudally tilted-up slice. The slices were placed in the recording chamber (volume, 1.0 ml), which was perfused with artificial cerebrospinal fluid (ACSF; in mM: 126 NaCl, 3 KCl, 1 MgCl_2_, 1.25 NaH_2_PO_4_, 26 NaHCO_3_, 2 CaCl_2_, and 10 d-glucose; pH 7.3) at a flow rate of 1.5 ml/min at room temperature (20–24 °C). ACSF was continuously gassed with 95% O_2_–5% CO_2_.

### Optical recording with a voltage-sensitive dye

The procedures for the optical recording and its data analysis have been described previously^42^. Briefly, the slices were stained with 200 μM RH414 (Invitrogen, Carlsbad, CA). The slices were illuminated with light of 535 ± 15 nm wavelength using a stabilized 150 W xenon lamp. The fluorescence emitted from voltage-sensitive dye was long-pass filtered above 580 nm and measured with a CCD camera (MiCAM02-HR, BrainVision, Tokyo, Japan), which was attached to a microscope (BX-51WI, Olympus, Tokyo, Japan) equipped with an air objective (XLFluor 4 × /340, 0.28 NA; Olympus). The imaged area was 4.6 × 3.1 mm^2^, and a 376 × 252 array detected the optical signals. A fluorescence image was captured every 8 ms for up to 2 s, yielding an optical response consisting of a series of up to 250 images. The optical data were analyzed by using BV_Ana (BrainVision), and were displayed as pseudocolor images. Each ROI has a diameter of 240 μm in which 325 pixels are included, and the signal amplitude at each ROI was the averaged value of the amplitudes of signals at respective pixels included in a ROI. As illustrated in Fig. 1a, ROI1 and ROI2 were placed in the regions of the Gu-I rostral to and facing MCA, respectively, and ROI3 was placed on the boundary between the Gu-I and GI-Au-I, ROI5 in the caudal end of the GI-Au-I, ROI4 in the middle point between ROI3 and ROI5, and ROI6 in the middle region of the CV-Au-I. The inter-center distance between respective ROIs was set to be larger than 400 μm, which is a diameter of a neuronal cell assembly represented as a column-like vertical excitation spread across all layers that is induced in the Gu-I by stimulation of dysgranular layer as shown previously^16^.

Before and 5 min after application of AEA or various blockers in the presence of AEA, a series of 30 optical responses were obtained by repeatedly capturing a series of 250 fluorescence images with a sampling rate of 125 Hz every 15 s. After confirming the absence of oscillatory optical responses in the control condition, AEA was bath-applied. When an optical recording was started 5 min after application of AEA, AEA-induced oscillatory optical responses were already ongoing. In the presence of CNQX (10 μM) and AP5 (50 μM), AEA did not induce any oscillation (Fig. S3g, n = 8). In the default mode of BV_Ana, the signal intensities (∆*F*/*F*_0_) at all pixels at a given timing during an ongoing propagating oscillation are aligned to be zero at the beginning of measurement (as the zero time point) in spite of the differences in the ∆*F*/*F*_0_ among all the pixels over slices due to the propagation delay and the differential inhibition (Fig. 6d). Such optical responses were reconstructed by aligning the mean values of the bottom peaks of oscillatory optical signals sampled every 8 ms for 2 s at the respective pixels to be zero (Fig. 6e).

Due to oscillatory changes in the signal intensity, the 1st frame of each optical response captured at time zero as the resting light intensity (*F*_0_) was not really a resting response. Furthermore, when the oscillation propagated into different regions, the apparent *F*_0_ may also be different among the respective pixel points at a given zero time during ongoing oscillation. However, as the ∆*F*/*F*_0_ that can be observed using RH414 is usually less than 1%, the *F*_0_ is at least 100 times larger than ∆*F* at all respective pixel points. Then, the possible difference in the apparent *F*_0_ between the given zero time points during oscillation is very small, and therefore might not affect the ∆*F*/*F*_0_ markedly at the respective pixel points. Therefore, the intensities of optical signals at given times are calculated as ∆*F*/*F*_0_. Because negative waves should be abolished by GABA receptor antagonists bicuculline and/or CGP, we regarded the bottom peak of the oscillatory optical responses in the presence of bicuculline and/or CGP (Fig. 6f) as the baseline level. Provided that there is no inhibitory action at the timing of the top peak of oscillatory waves, the waves obtained before and after application of bicuculline and/or CGP (blue and red traces, respectively) could be aligned with the top peaks of respective waves at ROIs1–6 (Fig. 6g) to determine the zero baseline level of the respective waves at ROIs1–6 obtained before bicuculline and/or CGP application. The inhibitory component may be underestimated by this method if there is any inhibitory action at the timing of the top peak. Therefore, the inhibitory component can be at least minimally dissected by this method. Subsequently, the pseudocolor images of optical responses obtained following AEA application were reconstructed by setting the baseline level in this manner (Figs 2b and 3d). To visualize the spatio-temporal profiles of the propagation of oscillatory optical responses along L3 from the Gu-I into the GI-Au-I, a line profile was constructed along a line drawn on L3 of slices (e.g. a pink solid line in Fig. 1a). The amplitudes of oscillatory optical signals at respective ROIs (3 × 3) under the line drawn along L3 measured every 8 ms for 1600 ms were represented as 201 series of 330–365 pseudocolor signals, which were aligned along the time (horizontal) and distance (vertical) axes and were subsequently represented as a pseudocolor image of a line profile.

Power spectral analysis^46^ of optical responses was performed using STATISTICA 10J (StatSoft, Truka, OK). Except in Fig. S3c,d, all PDs of the AEA-induced optical responses at ROIs2–6 and all PDs obtained at ROIs1–6 after application of testing solutions in the presence of AEA were normalized to the PD at 5 Hz of the control AEA-induced optical response at ROI1.

### Drug application

Anandamide (AEA; n = 76 and n = 5 slices obtained from Wistar and SD rats, respectively), ACEA (CB1 agonist; n = 4 slices obtained from Wistar rats) and AM251 (CB1 antagonist; n = 5 and n = 5 slices obtained from Wistar and SD rats, respectively) were bath-applied at 3, 1 and 10 μM, respectively. *N*-oleoylethanolamine (OEA; n = 15 slices obtained from Wistar rats), AR231453 (GPR119 agonist; n = 9 and n = 5 slices obtained from Wistar and SD rats, respectively), GW6471 (PPARα antagonist; n = 5 slices obtained from Wistar rats), GW7647 (PPARα agonist; n = 5 slices obtained from Wistar rats), arvanil (n = 5 slices obtained from Wistar rats) and 4-[3-(cyclopentyloxy)-4-methoxyphenyl]-2-pyrrolidinone (rolipram; n = 7 slices obtained from Wistar rats) were bath-applied at 10, 1, 10, 1, 50 and 10 μM, respectively. Bicuculline (n = 4 slices obtained from Wistar rats) and CGP55845 (n = 8 slices obtained from Wistar rats) were bath-applied both at 10 μM. Bicuculline at this concentration largely enhanced the optical responses evoked by stimulation of layer 4 by blocking GABA_A_Rs in the slice preparation^16^ which was the same as that used in the present study. 6-Cyano-7-nitroquinoxaline-2,3-dione (CNQX, a non-NMDA receptor antagonist) and dl-2-Amino-5-phosphonopentanoic acid (AP5, an NMDA receptor antagonist) were bath applied at 10 and 50 μM, respectively (n = 8 slices obtained from Wistar rats). ACEA, arvanil, CNQX and AP5 were obtained from Tocris Bioscience (Bristol, UK), and AR231453 and GW7647 were obtained from Abcam (Cambridge, MA, USA). All other chemicals were obtained from Sigma-Aldrich.

### Statistical analysis

Numerical data are expressed as the mean ± SD or as the geometric mean × [geometric SD]^±1^. Statistical significance was assessed using the unpaired (*) or paired (**) Student’s *t*-test or using one-way (^†^) or two-way (^‡^) ANOVA followed by Fisher’s PLSD (protected least significant difference) post hoc test. *p < *0.05 was considered statistically significant.

## Additional Information

**How to cite this article**: Kang, Y. *et al*. A role of CB1R in inducing θ-rhythm coordination between the gustatory and gastrointestinal insula. *Sci. Rep.* **6**, 32529; doi: 10.1038/srep32529 (2016).

## Supplementary Material

Supplementary Information

Supplementary Movie 1

Supplementary Movie 2

Supplementary Movie 3
