# Supplementary material for: A role of CB1R in inducing θ-rhythm coordination between the gustatory and gastrointestinal insula
Source: Sci Rep. 2016 Sep 1;6:32529. doi: 10.1038/srep32529 (PMC5007515; doi:10.1038/srep32529)
Supplement: Supplementary Information [file srep32529-s1.pdf]

Supplementary Information

A role of CB1R in inducing θ-rhythm coordination between the gustatory and gastrointestinal insula

Youngnam Kang, Hajime Sato, Mitsuru Saito, Dong Xu Yin, Sook Kyung Park, Seog Bae Oh, Yong Chul Bae and Hiroki Toyoda

Supplemental Methods

Whole-cell recordings

The procedures for the whole-cell recordings were previously described (Saito et al., 2012). The patch pipettes had a DC resistance of 4–5 MΩ when filled with the internal solution. The sealing resistance was usually more than 10 GΩ. The internal solution had the following composition (mM): 123 K-gluconate, 18 KCl, 14 NaCl, 2 ATP-Mg, 0.3 GTP-Na_3, 10 HEPES, 0.1 EGTA; pH 7.3, adjusted with KOH. Whole-cell currents were recorded with Axopatch 200B (Molecular Devices, Foster City, CA). Signals were low-pass filtered at 2 kHz (4-pole Bessel filter) and digitized at a sampling rate of 2–10 kHz (1440A, Molecular Devices). The membrane potential values given in the text were corrected for the liquid junction potential (10 mV) between the K-gluconate-based internal solution (negative) and the standard extracellular solution.

Electron microscopic immunohistochemistry

Because the Wistar and SD rats displayed similar oscillation in the Gu-I which is induced by activation of CB1Rs and abolished by activation of GPR119, immunohistochemical experiments were done only in SD rats to confirm the persistent presence of CB1R and GPR119 in the Gu-I even in 2–3 months old rats. Three male SD rats weighing 300–320 g were deeply anesthetized with sodium pentobarbital (80 mg/kg, i.p.) and perfused transcardially with 100 ml of heparinized normal saline followed by 500 ml of a freshly prepared mixture of 4% paraformaldehyde and 0.01% glutaraldehyde in 0.1 M phosphate buffer (PB; pH 7.4). The cerebral cortex, including insula between 2.0 and −0.8 mm from bregma, was removed and post-fixed in the same fixative for 2 h at 4°C. Sections were cut transversely on a Vibratome at 60 µm and cryoprotected in 30% sucrose in PB overnight at 4°C. Sections were frozen on dry ice for 20 min and thawed in phosphate buffered saline (PBS; 0.01 M, pH 7.2) to enhance penetration. They were pretreated with 1% sodium

borohydride for 30 min to remove glutaraldehyde and then blocked with 3% H_2O_2 for 10 min to suppress endogenous peroxidases and with 10% normal donkey serum (NDS; Jackson ImmunoResearch, West Grove, PA) for 30 min to mask secondary antibody binding sites.

For double immunostaining for CB1R or GPR119 and glutamic acid decarboxylase 65/67 (GAD65/67), sections containing insular cortex were incubated overnight in a mixture of goat anti-CB1 (1:50, sc-10068, Santa Cruz Biotechnology, Santa Cruz, CA) or rabbit anti-GPR119 (1:100, sc-99103, Santa Cruz Biotechnology) and mouse anti-GAD65/67 (1:1000, ADI-MSA-225-E, Enzo Life Sciences, Farmingdale, NY) antibodies. After rinsing in PBS, sections were incubated with a mixture of biotinylated donkey anti-goat or anti-rabbit (1:200, Jackson ImmunoResearch) and 1 nm gold-conjugated donkey anti-mouse (1:50, EMS, Hotfield, PA) antibody. The sections were post-fixed with 1% glutaraldehyde in PB for 10 min, rinsed in PB, incubated for 4 min with HQ silver enhancement solution (Nanoprobes, Yaphank, NY), and then rinsed in 0.1 M sodium acetate and PB. Sections were then incubated with ExtrAvidin peroxidase (1:5000, Sigma-Aldrich, St. Louis, MO) for 1 h, and the immunoperoxidase was visualized by nickel-intensified 3,3'-diaminobenzidine tetrahydrochloride. Sections were further rinsed in PB, osmicated (in 0.5% osmium tetroxide in PB) for 30 min, dehydrated in graded alcohols, flat embedded in Durcupan ACM (Fluka, Buchs, Switzerland) between strips of Aclar plastic film (EMS, Hatfield, PA), and cured for 48 h at 60°C. Chips in the superficial layer of the insular cortex were cut out of the wafers and glued onto blank resin blocks with cyanoacrylate. Serially-cut thin sections were collected on formvar-coated single-slot nickel grids and stained with uranyl acetate and lead citrate. Grids were examined on an electron microscope (H-7500; Hitachi High Technologies, Tokyo, Japan) at 80kV accelerating voltage. Images were captured with Digital Montage software driving a cooled CCD camera (SC1000; Gatan, Pleasanton, CA) attached to the microscope and were saved as TIFF files. To control for the specificity of primary antibodies, sections were processed as described above, except that blocking peptides were added at various concentrations. Specific immunostaining for GPR119 in the insular cortex was completely abolished by preabsorption with the antigen peptide at a final concentration of 35 µg/ml.

Supplemental Figures

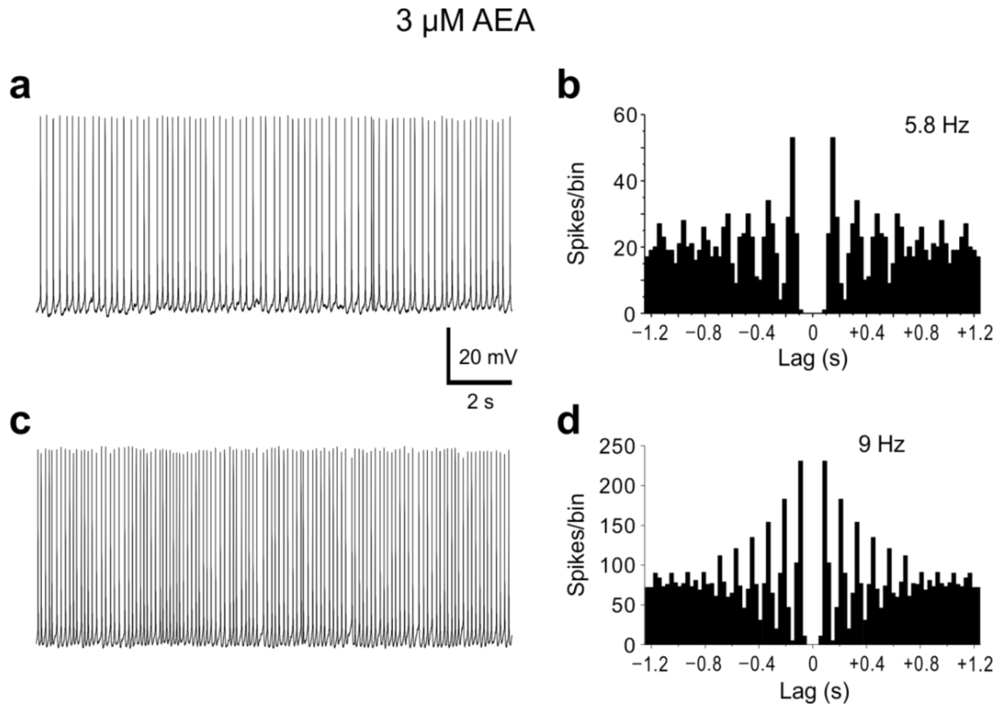

Figure S1. AEA induces sustained spike firings at θ-rhythm in L3 PCs of the Gu-I.

(**a**, **c**) Two representative examples.

(**b**, **d**) Autocorrelogram of the spike train (**a**) and that of the spike train (**c**) showing a periodicity of 5.8 Hz and that of 9 Hz, respectively. Bin width, 30 ms.

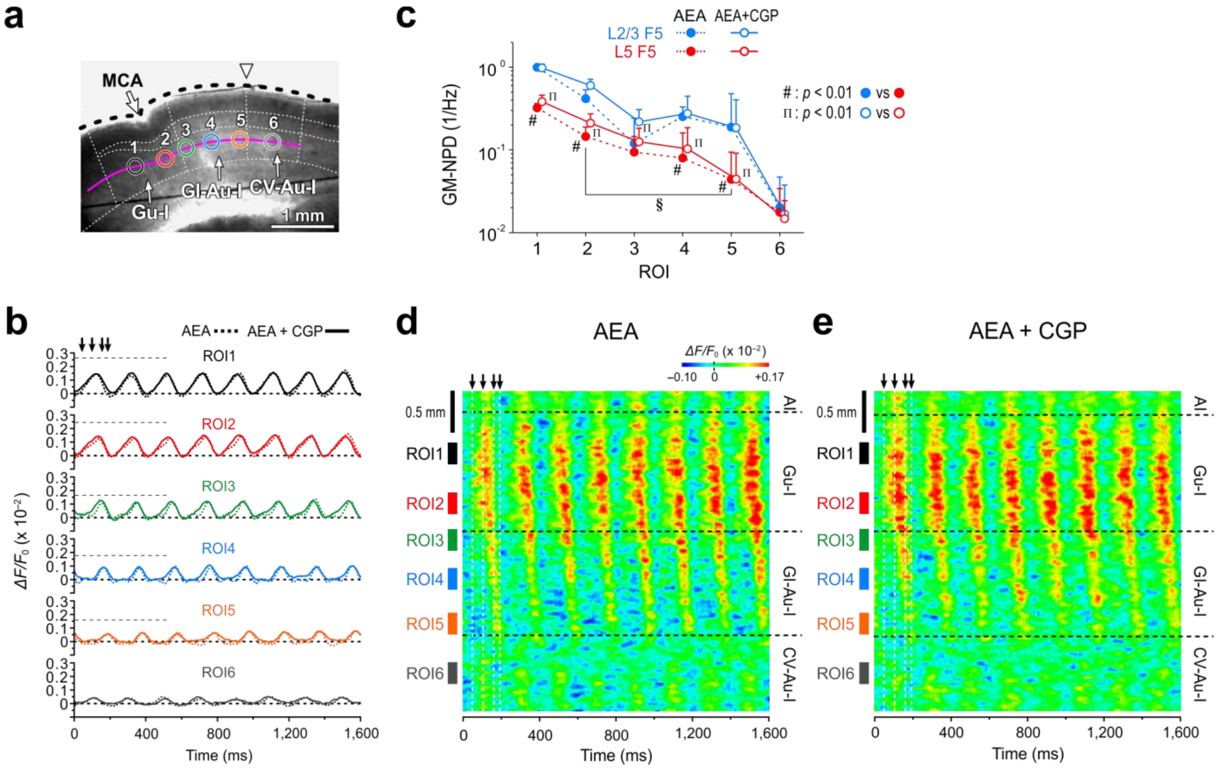

Figure S2. Poor propagation from the Gu-I to GI-Au-I in L5.

(**a**) A bright-field image of the insular cortex in the horizontal slice. The colored circles placed on a pink solid line drawn along L5 represent ROI1–6. ⇩: MCA. ∇: boundary between the GI-Au-I and CV-Au-I.

(**b**) Superimposed traces of the temporal profiles at ROI1–6 in L5 obtained before (dotted lines) and after application of 10 μM CGP in the presence of 3 μM AEA (solid lines). ↓: time points at which the respective pseudocolor images (Fig. 2**b**) were obtained. Horizontal interrupted lines at ROI1–5 show the top peak levels of the temporal profiles at ROI1–5 in L2/3. The amplitudes of optical responses in L5 were considerably smaller compared to those in L2/3.

(**c**) Filled red circles represent GM-F5-NPDs at ROI1–2 and ROI4–5 in L5, which were significantly ($^{‡}p < 0.01$) smaller compared to those in L2/3 represented by filled blue circles. Open red circles represent GM-F5-NPDs at ROI1–5 in L5 obtained following CGP55845 application, which were significantly ($^{‡}p < 0.01$) smaller compared to those in L2/3 represented by open blue circles. A significant difference was found in the GM-F5-NPDs between ROI2 and ROI5 ($^{‡}p < 0.01$) but not that between ROI2 and ROI4 ($^{‡}p > 0.1$) in L5 (filled red circles). GM-F5-NPDs in L2/3 and L5 following application of AEA alone: ROI1; 1 and $0.323 \times 1.1^{\pm 1}$, ROI2; $0.388 \times 1.4^{\pm 1}$ and $0.145 \times 1.3^{\pm 1}$, ROI3; $0.120 \times 2.0^{\pm 1}$ and

$0.094 \times 1.5^{\pm 1}$, ROI4; $0.279 \times 2.0^{\pm 1}$ and $0.080 \times 2.0^{\pm 1}$, ROI5; $0.189 \times 2.5^{\pm 1}$ and $0.044 \times 2.1^{\pm 1}$, ROI6; $0.020 \times 2.3^{\pm 1}$ and $0.018 \times 1.9^{\pm 1}$. Those following addition of CGP: ROI1; $0.983 \times 1.1^{\pm 1}$ and $0.385 \times 1.2^{\pm 1}$, ROI2; $0.587 \times 1.3^{\pm 1}$ and $0.211 \times 1.3^{\pm 1}$, ROI3; $0.219 \times 1.4^{\pm 1}$ and $0.126 \times 1.5^{\pm 1}$, ROI4; $0.276 \times 1.6^{\pm 1}$ and $0.103 \times 1.8^{\pm 1}$, ROI5; $0.186 \times 2.2^{\pm 1}$ and $0.044 \times 2.1^{\pm 1}$, ROI6; $0.017 \times 2.2^{\pm 1}$ and $0.015 \times 1.7^{\pm 1}$ (n = 5). $^{\ddagger(\#)}p < 0.01$. $^{\ddagger\,(\Pi)}p < 0.01$. $^{\ddagger\,(\S)}p < 0.01$.

(**d**, **e**) A line profile measured along L5 following application of AEA alone (**d**) and that following addition of CGP (**e**). There was no apparent difference in the spatial extent of excitation between the line profiles along L5 obtained before and after application of CGP55845.

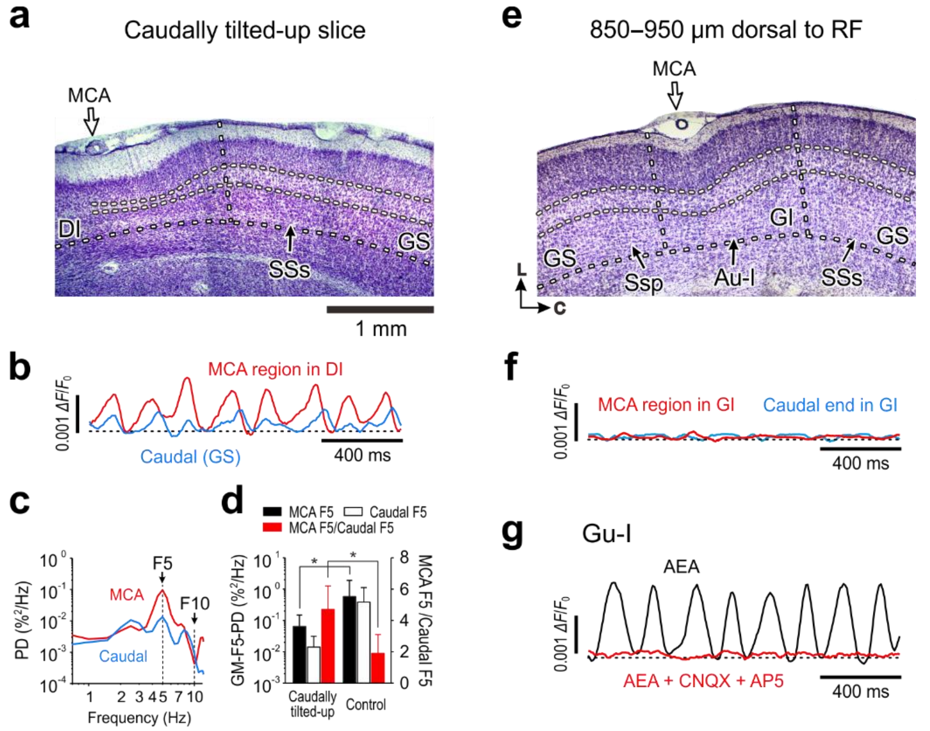

Figure S3. Optical responses induced by AEA in two different types of slices and abolishment of AEA-induced oscillation by CNQX and AP5.

(**a**) Nissl-stained histological section (100 μm thickness) of the caudally tilted-up slice of the insular cortex. The DI (Gu-I) was localized in the rostral one-third of such slices while the caudal two-third was mostly occupied by the somatosensory areas with a small area of GI-Au-I in its rostralmost part.

(**b**) Superimposed traces of the temporal profiles of AEA-induced responses at the MCA and caudal regions in a caudally tilted-up slice. AEA induced weak and irregular oscillation only in the region limited close to the MCA, and its propagation into the caudal region that presumably represents the secondary somatosensory cortex (SSs) was much more limited, compared to the control slices.

(**c**) The PDs of the responses, which were shown in **a**, at the MCA and caudal regions.

(**d**) GM-F5-PD in the MCA region was significantly smaller in the caudally tilted-up slices ($0.065 \times 1.3^{\pm 1}$ %^{2}/Hz, n = 5) than in the control slices ($0.435 \times 1.5^{\pm 1}$ %^{2}/Hz, n = 16) (compare black filled columns), presumably due to the smaller Gu-I region compared to the control. The ratio of F5-PD in the MCA region to that in caudal region (4.7 ± 1.5, n = 5) was significantly larger compared to the control (1.9 ± 1.2, n = 16) (compare red filled columns), suggesting the absence of positive feedback from the GI-Au-I to the Gu-I. *$p < 0.01$.

(**e**) Nissl-stained histological section (100 μm thickness) of the tilted horizontal slice at the level of 850–950 μm dorsal to the RF. The DI (Gu-I) was not included in the histological

section which instead included the granular sensory cortex (GS; SSp and SSs) and GI (Au-I).

(**f**) AEA induces neither oscillation nor propagation at the MCA region of the GI and at the caudal end of the GI in the slice cut at 750–1050 μm more dorsally from the RF.

(**g**) Sample traces of the temporal profiles of the AEA-induced responses in the Gu-I obtained before and after application of 10 μM CNQX and 50 μM AP5.

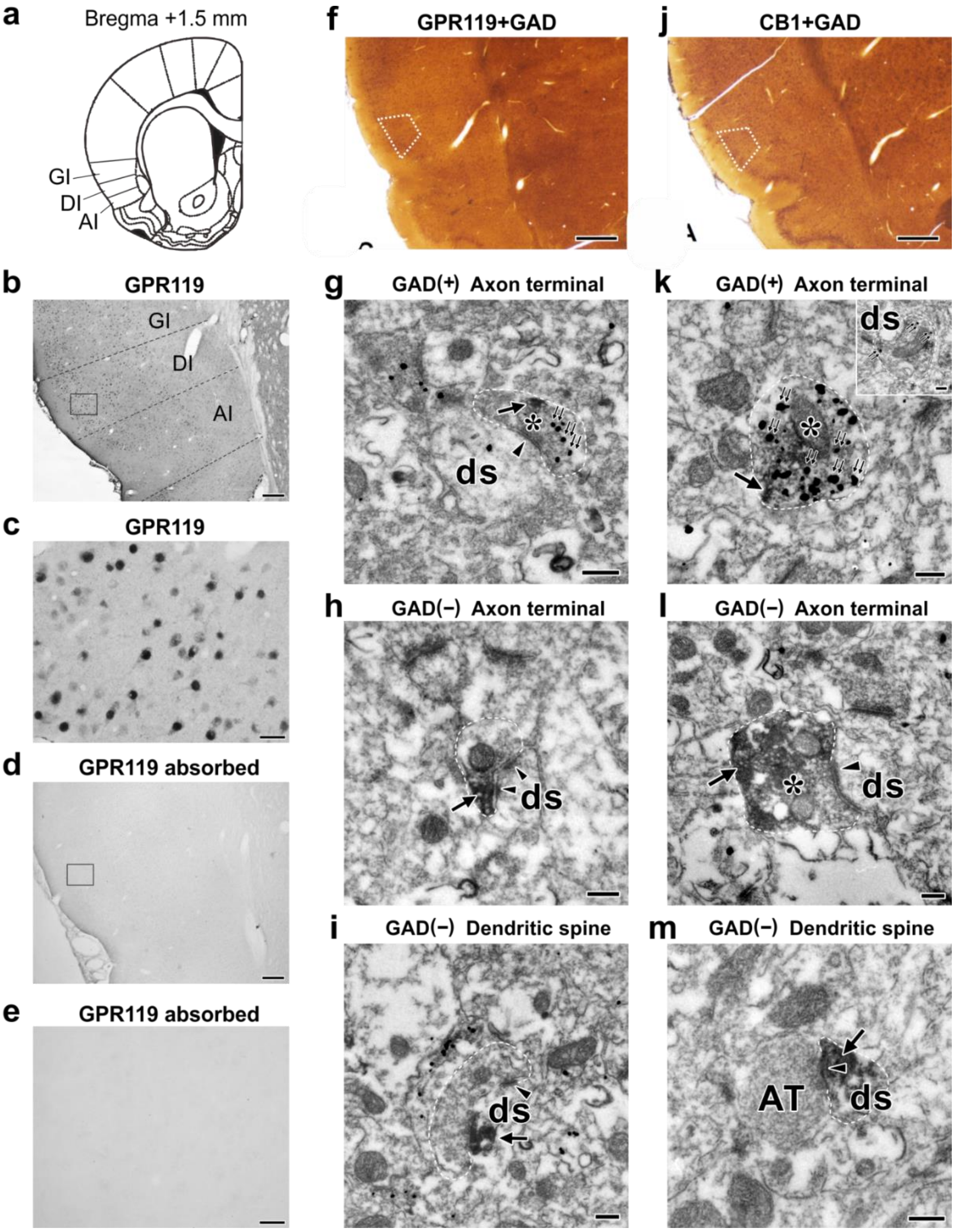

Figure S4. Subcellular distribution of the GPR119 and CB1R in the Gu-I.

(**a**) A tracing of a coronal brain section at +1.5 mm from the bregma showing subregions of the insular cortex.

(**b–e**) Light micrographs showing immunostaining for GPR119 in the Gu-I (**b**, **c**). The immunostaining with rabbit anti-GPR119 antibody was completely abolished by preabsorption with corresponding blocking peptide (35 µg/ml) (**d**, **e**). (**c**) and (**e**) are enlargements of the boxed areas in (**b**) and (**d**), respectively. Scale bars = 200 µm in (**b**) and (**d**), 20 µm in (**c**) and (**e**).

(**f**, **j**) Bright-field images of coronal sections of the Gu-I at +1.5 mm from the bregma, in which double immunostaining for CB1R and GAD65/67 or for GPR119 and GAD65/67 was performed (**f** and **j**, respectively). Electron microscopic examination was performed in the trapezoidal enclosed areas (dotted lines) shown in the superficial layer (L2/3) of Gu-I. Scale bar, 500 µm.

(**g**–**i**) Electron micrographs of the Gu-I at +1.5 mm from bregma showing that CB1R was expressed in the axon terminals of GABAergic neurons (**g**) as well as non-GABAergic neurons (**h**) and in the dendritic spines of non-GABAergic neurons (**i**). Double arrows: GAD-positive puncta. Arrows: CB1R-positive puncta. Arrowheads: site of synaptic contact. Scale bar, 200 nm.

(**k**–**m**) Electron micrographs of the Gu-I at +1.5 mm from bregma, showing that GPR119 was expressed in the axon terminals of GABAergic neurons (**k**) as well as non-GABAergic neurons (**l**) and in the dendritic spines of non-GABAergic neurons (**m**). Double arrows: GAD-positive puncta. Arrows: GPR119-positive puncta. Arrowheads: site of synaptic contact. Scale bar, 200 nm.

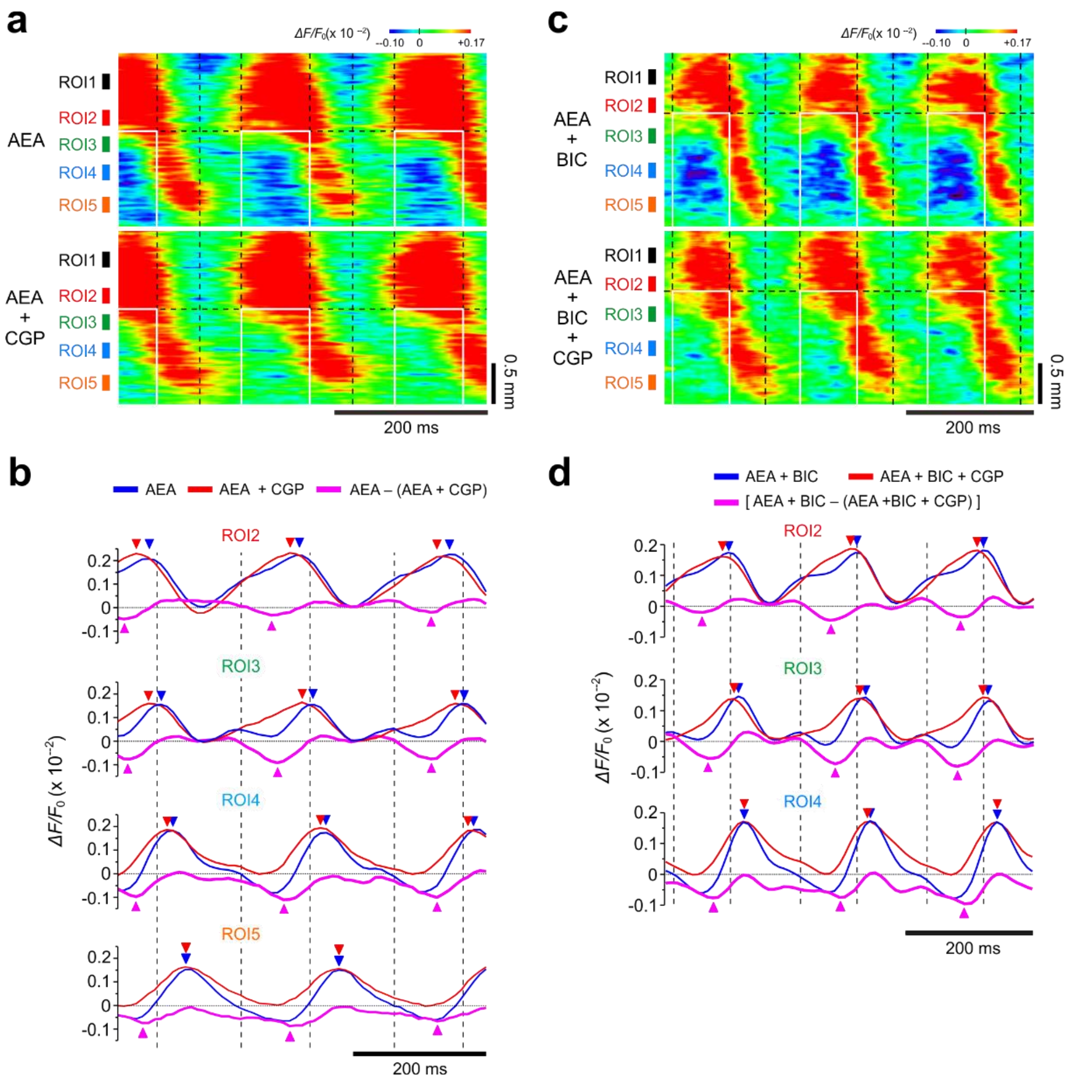

Figure S5. GABA$_B$R-mediated feed-forward lateral inhibition.

(**a**, **c**) The line profiles seen in the rectangle areas in Fig. 2**c** and **d** and those in Fig. 3**f** and **g** were re-illustrated on the expanded time scale (**a** and **c**, respectively) together with the corresponding temporal profiles (**b** and **d**, respectively).

Supplemental Movie Legends

Movie S1. AEA-induced neural coordination between the Gu-I and GI-Au-I.

Following application of AEA, excitatory optical responses emerged in the Gu-I and propagated caudally into the GI-Au-I, which was repeated periodically at 5 Hz. The oscillatory excitation did not invade into the CV-Au-I. ∇: boundary between the Gu-I and GI-Au-I. A black interrupted line shown in the trans-illuminated image: pia mater. No baseline correction was made.

Movie S2. AEA-induced neural coordination between the Gu-I and GI-Au-I obtained after resetting the baseline level of the optical response.

The AEA-induced optical response was corrected by regarding the CGP-sensitive component as a negative component. The inhibitory optical responses emerged in the GI-Au-I prior to the propagation of the excitatory optical responses into the GI-Au-I. ∇: boundary between the Gu-I and GI-Au-I. A black interrupted line: pia mater.

Movie S3. Effects of CGP55845 on the AEA-induced neural coordination between the Gu-I and GI-Au-I.

Following the addition of CGP55845 to the bath solution containing AEA, the propagation of the excitatory optical responses from the Gu-I to the GI-Au-I was facilitated compared to the control responses (Movie S2). Nevertheless, the oscillatory excitation did not invade into the CV-Au-I. ∇: boundary between the Gu-I and GI-Au-I. A black interrupted line: pia mater.
